# The mediating role of externalising and healthy schema modes in the relationship between early maladaptive schemata and overt behaviours in adolescent boys with offending behaviours, and a comparison of their early schemata with those of typically developing boys

**DOI:** 10.1002/cbm.2192

**Published:** 2021-03-25

**Authors:** Dorien L. C. Schilder, Marjolein F. van Wijk‐Herbrink, Annabeth P. Groenman, Barbara J. van den Hoofdakker

**Affiliations:** ^1^ Department of Child and Adolescent Psychiatry University Medical Center Groningen University of Groningen The Netherlands; ^2^ VIGO Group Zetten The Netherlands; ^3^ Department of Psychology Autism & ADHD Research Center (d'Arc), Brain and Cognition University of Amsterdam The Netherlands; ^4^ Department of Clinical Psychology and Experimental Psychopathology University of Groningen The Netherlands

**Keywords:** adolescents, early maladaptive schemata, externalising behaviour problems, schema modes

## Abstract

**Background:**

Evidence‐based treatments in routine clinical practice often fail to achieve or sustain amelioration of severe behaviour problems in adolescents. Better understanding of mechanisms underlying such severe behaviour problems could improve treatments. Underlying schemata and schema modes may play an important role.

**Aims:**

To compare early maladaptive schemata, schema modes and behaviour problems in adolescent boys showing disruptive and offending behaviours with those in typically developing boys. We hypothesised a relationship between disconnection and rejection schemata on the one hand and behaviour problems (including offending) on the other in adolescent boys with disruptive behaviour disorders. We also hypothesised that this offending group would differ significantly from typically developing boys on these measures and that schema modes would mediate relationships between schemata and overt behaviours.

**Method:**

In this cross‐sectional study, fifty‐five 12–19‐year‐old boys with disruptive behaviour disorders referred to an in‐ or out‐patient clinic were matched to fifty‐five typically developing boys from a previously generated school sample. Group differences on self‐reported schema related measures and externalising behaviour measures were compared using *t*‐tests. Mediation analyses were performed to assess the mediating role of schema modes in the relation between schemata and behaviour.

**Results:**

Boys diagnosed with disruptive behaviour disorders and engaging in offending behaviours had higher scores on externalising modes and lower scores on healthy modes than the typically developing boys. There were no differences between these groups, however, in disconnection and rejection schemata. In the offending behaviour group, externalising modes mediated the relationship between *disconnection and rejection schemata* and externalising behaviours while healthy modes mediated a relationship between these schemata and overt prosocial behaviours.

**Implications:**

The potential impact of healthy modes has not previously been shown in studies of schemata in young offenders. Our findings suggest that treatments for adolescents with severe behaviour problems should not only target maladaptive schemata and dysfunctional modes, but seek also to boost healthy modes.

## 1 INTRODUCTION

Adolescents with severe behaviour problems are at high risk of developing persistent offending behaviours (Barrett et al., 2014; Byrd et al., 2012), comorbid disorders and psychosocial problems (Cohn & Adesman, 2015; Kazdin, 2015). Despite the existence of evidence‐based multicomponent interventions for the reduction of conduct problems in this age group, treatment in routine clinical practice often fails to achieve or retain substantial improvements (Dekovic & Stoltz, 2015; Greenwald, 2009; Masi et al., 2013). Therefore, gaining more insight in the mediating mechanisms that are associated with severe behaviour problems is important, as this may facilitate the development and evaluation of new, better tailored, treatments (Kazdin, 2007). Schema theory provides a model for comprehending the relationship between early stable cognitive and emotional organisational processes—or schemata—and observed behaviours and changing behavioural patterns (Young et al., 2003). Early maladaptive schemata and schema modes are central constructs in schema theory, defined as maladaptive mental representations of oneself and one's connection with others. Schema theory considers that such schemata develop early in life, probably as a result of the interaction between a child's temperament and adverse childhood experiences. There is evidence of their long‐term stability (Riso et al., 2006) and they may become pervasive as the person develops, because of their self‐perpetuating nature. Schemata associated with attachment are thought to develop most early in life from experiences related to disconnection and rejection. Disconnection and rejection schemata, such as *abandonment*—the expectation that one will be abandoned by significant others—or *mistrust*—the expectation that one will be harmed by others—distort the processing of social information and arouse negative emotions. Schema theory assumes that coping reactions to activation of such trait‐like schemata result in schema modes, which are overt states that dominate one's emotions, cognitions and actions. Schema modes may be classified as internalising, externalising, or healthy (Keulen‐De‐Vos et al., 2017; Van Wijk‐Herbrink, Roelofs et al., 2017). Internalising modes may be associated with vulnerability (e.g., Abandoned Child mode), self‐punishment (e.g., Punitive Parent mode) or excessive adjustment to others (e.g., Compliant Surrenderer mode). Externalising schema modes are modes associated with emotions such as anger (e.g., in Angry Child mode) or actions such as lack of self‐control or self‐discipline (e.g., in Undisciplined Child mode) or aggression (e.g., in Bully and Attack mode). Healthy modes, on the other hand, incorporate functional states of spontaneity (e.g., in Happy Child mode) and self‐compassion and self‐care (in Healthy Adult or Healthy Adolescent mode). Conversely, learning to cope adequately with early maladaptive schemata will result in healthy schema modes that may predispose to prosocial behaviour (Young et al., 2003).

Schema therapy may be a promising treatment for changing externalising behaviour problems (Young et al., 2003). Previous research with adult offender patients with externalising behaviours and personality disorders indicates that schema therapy can be an effective treatment for reducing recidivism risk and promoting re‐entry into the community (Bernstein et al., 2021). A recent study found preliminary evidence for the effectiveness of schema therapy with adolescents with externalising behaviour problems (Van Wijk‐Herbrink et al., 2017). Given these promising findings, gaining more insight into underlying mechanisms may give further direction to treating such problems more effectively.

A few studies with adolescents have found that behaviour problems were positively associated with early maladaptive schemata (Van Vlierberghe et al., 2010; Van Wijk‐Herbrink et al., 2018) and externalising schema modes (Van Wijk‐Herbrink, Roelofs et al., 2017). Furthermore, there is some evidence that externalising schema modes mediate the relation between early maladaptive schemata and externalising behaviours (Van Wijk‐Herbrink et al., 2018). By contrast, healthy schema modes may be associated with less psychopathology (Roelofs et al., 2016). At the time of writing, however, no study had examined the role of healthy schema modes in the relationship between early maladaptive schemata and overt behaviours.

Our aim was to compare early maladaptive schemata between adolescent boys with offending behaviours and adolescent boys from a community sample, and to examine relationships between early maladaptive schemata, externalising schema modes, healthy schema modes and overt behaviours among adolescent boys with severe behaviour problems. We hypothesised that boys in the offending behaviour group would have higher levels of disconnection and rejection schemata and externalising schema modes, and lower levels of healthy schema modes, compared to boys in the community group. Moreover, in the offending behaviour group, we aimed to investigate the mediating role of schema modes in any relationship between disconnection and rejection schemata on the one hand and overt behaviours on the other. We hypothesised that externalising schema modes would mediate any relationship between disconnection and rejection schemata and externalising behaviours, and that healthy schema modes would mediate the relation between these schemata and prosocial behaviours.

## 2 METHOD

The medical ethical committee of the University Medical Center Groningen (UMCG) has appraised this study as not subject to the Dutch Law of Medical Scientific Research (registered in the UMCG research register, reference number 201600178).

### 2.1 Participants

Eligibility for the offending behaviour group was determined by presence of all the following inclusion criteria: (1) being male; (2) between 12 and 23 years old; (3) meeting criteria for oppositional defiant disorder, conduct disorder or disruptive behaviour disorder not otherwise specified according to the fourth edition of the American Diagnostic and Statistical Manual (DSM‐IV; American Psychiatric Association, 2000) as confirmed by an experienced psychologist, who had completed a post‐master programme in healthcare psychology, which includes clinical training and training in the use of DSM‐IV) and (4) having committed a criminal act, regardless of police or justice involvement (based on self‐report or patient record). Exclusion criteria were (1) the presence of acute mental health problems which required an immediate intervention (e.g., suicidality, severe self‐harm), as assessed by the clinician who was involved in the first diagnostic evaluation of the adolescent; (2) insufficient mastery of the Dutch language and (3) insufficient cognitive abilities to fill out the questionnaires, as estimated by a clinician.

### 2.2 Procedure

The offending behaviour group was recruited from two Dutch child and adolescent mental health centres, providing both in‐and outpatient care for adolescent boys with severe behaviour problems. All 12–23‐year‐old males who had been referred to outpatients within the previous 3 months or was resident in one of the centres were invited to participate in our study by the clinician who was involved in the initial diagnostic evaluation. After the young person and his carer(s) with legal authority had given informed consent, he was invited to fill out three questionnaires in the period before his treatment started, and received a voucher of €10 after their completion.

The control group was of a same‐size sample of sex‐, age‐ and nationality matched boys from a community sample recruited from a secondary school in the Netherlands. The larger community sample was from an earlier study, detailed elsewhere (Van Wijk‐Herbrink et al., 2017).

### 2.3 Measures

We used the 25 items from the disconnection and rejection domain of the self‐report Young Schema Questionnaire for Adolescents (YSQ‐A; Van Vlierberghe et al., 2004; Young & Brown, 1990) to measure the maladaptive schemata associated with disconnection and rejection experiences (Lee et al., 1999; Muris, 2006; Schmidt et al., 1995; Van Vlierberghe et al., 2010); these are: *abandonment* (expecting to be abandoned in intimate relations); *mistrust/abuse* (expecting to be mistreated by others); *emotional deprivation* (expecting to be deprived in one's emotional needs); *social isolation* (perceiving oneself as different from others) and *defectiveness/shame* (perceiving oneself as inferior). Each early maladaptive schema consists of five items (e.g., ‘I feel that people will take advantage of me’; ‘I don't fit in’) to be rated on a 6‐point Likert scale (1 = *not at all true* through to 6 = *totally true*). The YSQ‐A has the same items as the Young Schema Questionnaire‐Short Form (YSQ‐SF) for adults (Young & Brown, 1998), except for some language adaptations to suit adolescents. The YSQ has been found to have sound psychometric properties in adolescents (Muris, 2006; Van Vlierberghe et al., 2010). Higher scores indicate more severe early maladaptive schemata.

We used the short version (80‐item) of the self‐report Schema Mode Inventory (SMI; Lobbestael et al., 2010) to measure externalising and healthy schema modes. Previous studies (Keulen‐De‐Vos et al., 2017; Van Wijk‐Herbrink et al., 2017) have confirmed that externalising schema modes may be labelled as Angry Child (feeling and expressing anger in an excessive way), Enraged Child (losing control over angry feelings and attacking), Impulsive Child (acting impulsively to get what one needs), Undisciplined Child (acting like a spoiled child) and Bully and Attack mode (using unacceptable behaviour to get what one wants). Healthy modes have been labelled as Healthy Adult (or Healthy Adolescent; having awareness of one's needs and feelings, and moderating, nurturing or healing other modes) and Happy Child (experiencing pleasure and acting playful and carefree as core emotional needs are met). In the present study, we used the mean score on the Externalising and Healthy modes. Each schema mode consists of five items (e.g., ‘I have rage outbursts’; ‘I feel optimistic’) to be rated on a 6‐point Likert scale (1 = *never or hardly ever* through to 6 = *always*). Higher scores indicate a stronger presence of schema modes. Studies have shown that the SMI has adequate psychometric properties with adolescents (Roelofs et al., 2016; Van Wijk‐Herbrink et al., 2017).

Externalising and prosocial behaviours were measured with the Dutch version of the Strengths and Difficulties Questionnaire (SDQ; Goodman, 1997; Van Widenfelt et al., 2003), a self‐reporting screening instrument for social and emotional well‐being in children and adolescents. The subscales *conduct problems* (externalising behaviours) and *prosocial behaviour* each consist of five items, each with three categories (0 = *not true,* 1 = *somewhat true,* 2 = *certainly true*). Higher scores indicate stronger presence of that behaviour. The SDQ has been shown to have satisfactory psychometric properties (Muris et al., 2003; Van Widenfelt et al., 2003).

### 2.4 Statistical analyses

We analysed our data using Statistical Package for the Social Sciences (SPSS version 25). We performed independent samples *t*‐tests to assess differences in disconnection and rejection schemata, externalising modes and healthy modes between the two groups using a bootstrapping procedure (based on 1000 bootstrap samples) because the assumptions of normality and/or homoscedasticity were violated. Cohen's *d* (Cohen, 1977) was used to calculate effect sizes of the differences in means between the offender and typical community group on the schema modes and early maladaptive schemata. To calculate the sample size required, we used the G*Power 3 program (Faul et al., 2007). We found that a sample size of 51 participants per group would be needed to detect a medium effect at a 0.05 significance level while reaching 80% power.

We performed mediation analyses to assess whether externalising schema modes mediated any relationship between disconnection and rejection schemata and externalising behaviours, and to assess whether healthy schema modes mediated the relation between these schemata and externalising behaviours and prosocial behaviours. These mediation analyses were performed only in the offending behaviour group, because in the community group behaviours were assessed with a different measure. For the mediation analyses we used PROCESS macro (Hayes, 2013), which is based on ordinary least squares regression analysis, and calculated bias‐corrected bootstrap confidence intervals (based on 50,000 bootstrap samples). We used the completely standardised indirect effect (CSE; Field, 2013; Hayes, 2013; Preacher & Hayes, 2008) as a measure of the effect size of the indirect effect. We used the MedPower program (Kenny, 2017) to establish the power of the test of joint significance of paths a and b in the mediation analyses (MacKinnon et al., 2002). The result of a MedPower analysis can be considered as an estimate of the power of the mediation analysis, as the bootstrapping method has been found to have more power than the joint significance test to find indirect effects (Fritz, Taylor & MacKinnon, 2012). The MedPower analysis demonstrated that a sample size of 59 is sufficient to have 80% power of detecting a significant effect at a 0.05 level, with paths a and b having regression coefficients of 0.40. Due to the bootstrapping procedure and proximity of our sample size to this figure, we judged that there should be no further concerns about power.

## 3 RESULTS

### 3.1 General characteristics of the samples

The 55 adolescent boys were all between 12 and 19 years old (mean [*M*]: 15.49 years, standard deviation [*SD*]: 1.68). Most (52, 95%) were of Dutch nationality. Most of the boys (39, 71%) were attending (pre)vocational secondary education (which means that they were not being taught for academic qualifications but were learning practical skills with a view to taking up a trade such as furniture building or plumbing). Of the remaining boys, 5 (9%) attended higher general secondary education, while 11 boys (20%) did not attend any school at time of referral. Of the latter group, 10 boys (91%) had attended (pre)vocational education and the 1 remaining boy had attended general secondary education before dropping out from school. At the time of our assessments, 20 boys (38%) were staying at a youth care facility, of whom 12 were in a secured setting. Fourteen boys (26%) were living with their biological mother, 12 (22%) were living with both biological parents, 7 (13% with meaningful others and 2 (4%) with their biological father.

The sex‐, age‐ and nationality‐matched community group consisted of 55 boys who were between 12 and 18 years old (*M* = 15.44; *SD* = 1.61). All were living with one or both parents.

The descriptive statistics for the *disconnection* and *rejection schemata*, externalising schema modes, and healthy schema modes are displayed in Table 1. The offending behaviour and typically developing groups did not differ in *disconnection and rejection* schemata (*t*(101.47) = 0.66; *p* = 0.51; *d* = 0.13; 95% BCI = −0.17 to 0.33). The groups differed significantly on externalising modes (*t*(92,08) = 4.17; *p* < 0.001; *d* = 0.80; 95% BCI = 0.35–0.90) and healthy schema modes (*t*(108) = −1.98; *p* = 0.05; *d* = 0.38; 95% BCI = −0.70 to −0.01).

**TABLE 1:** Descriptive statistics for the *disconnection and rejection schemata*, externalising schema modes and healthy schema modes in the offending behaviour group and typically developing boys

Study variable	Offending behaviour group		Typically developing group	
	*M*	*SD*	*M*	*SD*
Disconnection and rejection schemata	2.03	0.11	1.94	0.08
Abandonment	1.99	1.06	2.20	0.82
Mistrust/abuse	2.43	1.18	1.69	0.73
Emotional deprivation	2.00	0.87	2.30	0.91
Social isolation	1.95	0.93	1.91	0.85
Defectiveness/shame	1.76	0.78	1.58	0.61
Externalising schema modes	2.46	0.13	1.82	0.08
Angry child	2.36	1.13	1.81	0.81
Enraged child	2.43	1.31	1.56	0.72
Impulsive child	2.72	1.19	1.85	0.83
Undisciplined child	2.62	1.02	2.20	0.78
Bully and attack	2.16	1.18	1.70	0.66
Healthy schema modes	4.24	0.14	4.59	0.12
Healthy adult	4.59	1.13	4.67	0.91
Happy child	3.88	1.05	4.51	1.02

Abbreviations: *M*, mean; *SD*, standard deviation.

**FIGURE 1:**
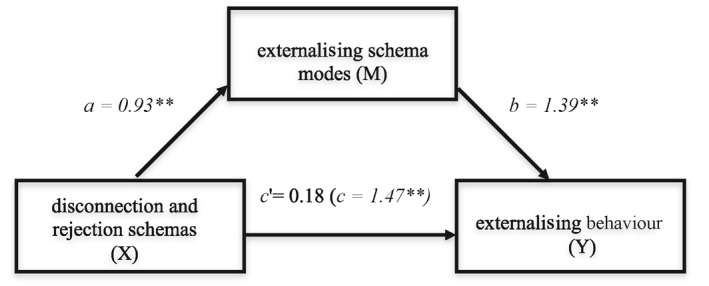
Simple mediation model estimating the direct and indirect effect of disconnection and rejection schemas on externalising behaviour. **Significant at the 0.01 significance level

**FIGURE 2:**
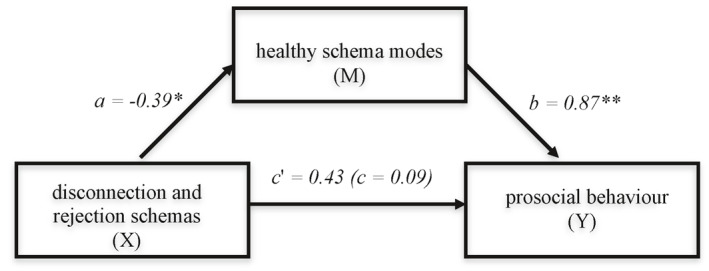
Simple mediation model estimating the direct and indirect effect of disconnection and rejection schemas on prosocial behaviour. *Significant aht the 0.05 significance level. **Significant at the 0.01 significance level

Our mediation model assessing whether externalising schema modes mediated the relationship between *disconnection and rejection* schemata and externalising behaviours showed a significant indirect effect (*ab*) of *disconnection and rejection* schemata on externalising behaviour through externalising modes (path *ab* = 1.29; BCI = 0.63–2.08) (see Figure 1 and Table 2). These schemata were associated with externalising modes (path a) which were, in turn, associated with externalising behaviours (path b), with a large effect size (CSE = 0.45; 95% BCI = 0.23–0.72). Path c (total effect) shows a significant relationship between *disconnection and rejection schemata* and externalising behaviours. This relationship disappears (path c'; direct effect) when controlling for path ab (path c' = 0.18; 95% BCI = −0.77 to 1.13). Thus, externalising schema modes mediated the relationship between *disconnection and rejection schemata* and externalising behaviours. The mediation model is displayed in Figure 1, The second mediation model examined whether healthy modes mediated the relationship between disconnection and rejection schemata and prosocial behaviours (Figure 2 and Table 2). We found a significant indirect effect of *disconnection and rejection schemata* on prosocial behaviours through healthy modes (path ab = −0.34, BCI = −0.73 to −0.05). These schemata were negatively associated with healthy modes (*a*). Healthy modes were associated with prosocial behaviours (*b*). The effect size was medium (CSE = −0.15; 95% BCI = −0.30 to −0.02). We found no evidence for a direct effect of early maladaptive schemata on prosocial behaviours (c' = 0.43; 95% BCI = −0.15 to 1.01). Thus, healthy schema modes mediated the relation between disconnection and rejection schemata and prosocial behaviours. The mediation model is displayed in Figure 2, and the results of the mediation analysis can be found in Table 2.

**TABLE 2:** Results of mediation analyses investigating the role of externalising versus healthy schema modes in relationships between disconnection and rejection schemata and externalising behaviours or prosocial behaviours

	Direct effect			Indirect effect		
	*R* ^2^	Effect	*SE*	Effect	*SE*	CSE
X = DR, M = EM, Y = EB	0.41	0.18	0.47	1.29	0.37	0.45
X = DR, M = HM, Y = PB	0.22	0.43	0.29	−0.34	0.17	−0.15

Abbreviations: CSE, effect size of the indirect effect; DR, disconnection and rejection schemata; EB, externalising behaviours; EM, externalising modes; HM, healthy modes; PB, prosocial behaviours; *R* ^2^, proportion of explained variance in the model with schema and modes as potential predictors.

## 4 DISCUSSION

We found evidence for a higher level of externalising schema modes and a lower level of healthy schema modes in our offending behaviour group compared to the typically developing boys. No differences, however, were found in *disconnection and rejection schemata*. In the offending behaviour group we found a mediating role of both externalising and healthy schema modes in the relationship between *disconnection and rejection schemata* and behaviours. Healthy modes mediated a relationship between the same schemata and prosocial behaviours.

The results of our mediation analyses were in line with the findings of a recent study from our group (Van Wijk‐Herbrink et al., 2018), indicating that *disconnection and rejection schemata* are linked to externalising behaviours through activated states of anger, impulsivity and indiscipline. *Disconnection and rejection schemata* are primary schemata related to adverse attachment experiences and trauma and have been proposed as key to the understanding of the development and persistence of externalising behaviour problems (Greenwald, 2002; Schimmenti et al., 2014). The activation of *disconnection and rejection schemata* is associated with intense feelings of loss, mistrust, failure and fear. The adoption of an overcompensatory coping style, which is a typical feature of externalising modes, to deal with these feelings, may result in oppositional and violent behaviours (Van Wijk‐Herbrink et al., 2018). These behaviours, in turn, may elicit dismissive or aggressive responses in others, essentially affirming the *disconnection and rejection schemata* of the adolescent. Hence, ameliorating attachment and trauma related problems in treatment may help to break this vicious cycle (Greenwald, 2002).

Our study also suggests that another way to treat adolescents with externalising behaviour problems is to teach them healthy coping responses (as a feature of healthy modes) so that they can deal with *disconnection and rejection schemata*, and be more likely to engage in prosocial behaviours. This supports a recent trend to integrate principles of positive psychology in schema therapy (Lockwood & Perris, 2012; Louis et al., 2018), building on personal strengths and enhancing positive experiences with others. These positive experiences could serve as an antidote for *disconnection and rejection schemata* (Young et al., 2003). Treatments that foster development of strategies both for overcoming adverse childhood experiences and stimulating healthy coping rather than ‘overcompensatory coping’, may be most effective in reducing externalising behaviour problems.

One finding of our study was counterintuitive: the offending behaviour group did not show higher levels of *disconnection and rejection schemata* than the typically developing boys in the community. It is possible that the adolescents in the offending behaviour group were responding in a pattern that has been referred to as ‘super normality’ (Cima et al., 2003), showing a social desirability response bias that has previously been found in other offender populations (Cima et al., 2003; Keulen‐de‐Vos et al., 2011; Lobbestael et al., 2009).

The largest strength of the study we report here is that, to the best of our knowledge, it is the first in which the role of healthy modes and prosocial behaviours have been examined in relation to externalising behaviours in adolescents. Inevitably, however, there are also limitations. First, our cross‐sectional design does not allow us to make any inferences about causality of the associations. Our assumptions were based on the schema theoretical model. Further research is needed to clarify and confirm the direction of the effects. Second, we relied solely on self‐report measures, possibly yielding response bias. In future research, self‐report measures should be combined with reports from significant others and/or clinical observations. In addition, we cannot rule out the possibility that inpatient boys from the offending behaviour group had already started treatment at the time of the study, possibly affecting their scores on the outcome measures.

Our findings support the importance of assessing schema modes and early maladaptive schemata in adolescent boys with severe behaviour problems, particularly anger and whether such feelings are connected to early childhood experiences in the absence of secure relationships. Our findings, however, go further in suggesting the importance of the potentially ameliorating effects of positive schema modes. Together these findings suggest a new direction for interventions, not only tailoring them to reduce negative internalised models of self, but also enhancing more positive schemata and coping styles.

## Data Availability

The data that support the findings of this study are available from the corresponding author, MvW, upon reasonable request.
